# ProFeatMap: a highly customizable tool for 2D feature representation of protein sets

**DOI:** 10.1093/bioadv/vbad022

**Published:** 2023-03-09

**Authors:** Goran Bich, Elodie Monsellier, Gilles Travé, Yves Nominé

**Affiliations:** Equipe Labellisée Ligue 2015, Department of Integrated Structural Biology, Institut de Génétique et de Biologie Moléculaire et Cellulaire (IGBMC), CNRS UMR 7104, INSERM U1258, Université de Strasbourg, Illkirch 67404, France; Equipe Labellisée Ligue 2015, Department of Integrated Structural Biology, Institut de Génétique et de Biologie Moléculaire et Cellulaire (IGBMC), CNRS UMR 7104, INSERM U1258, Université de Strasbourg, Illkirch 67404, France; Equipe Labellisée Ligue 2015, Department of Integrated Structural Biology, Institut de Génétique et de Biologie Moléculaire et Cellulaire (IGBMC), CNRS UMR 7104, INSERM U1258, Université de Strasbourg, Illkirch 67404, France; Equipe Labellisée Ligue 2015, Department of Integrated Structural Biology, Institut de Génétique et de Biologie Moléculaire et Cellulaire (IGBMC), CNRS UMR 7104, INSERM U1258, Université de Strasbourg, Illkirch 67404, France

## Abstract

**Motivation:**

Studies of sets of proteins are a central point in biology. In particular, the application of omics in the last decades has generated lists of several hundreds or thousands of proteins or genes. However, these lists are often not inspected globally, possibly due to the lack of tools capable of simultaneously visualizing the feature architectures of a large number of proteins.

**Results:**

Here, we present ProFeatMap, an intuitive Python-based website. For a given set of proteins, it allows to display features such as domains, repeats, disorder or post-translational modifications and their organization along the sequences, into a highly customizable 2D map. Starting from a user-defined protein list of UniProt accession codes, ProFeatMap extracts the most important annotated features available for each protein from one of the well-established databases such as Uniprot or InterPro, allocates shapes and colors, potentially depending on quantitative or qualitative data and sorts the protein list based on homologous feature content. The resulting publication-quality map allows even large protein families to be explored, and to classify them based on shared features. It can help to gain insights, for example, feature redundancy or feature pattern, that were previously overlooked. ProFeatMap is freely available on the web at: https://profeatmap.pythonanywhere.com/.

**Availability and implementation:**

Source code is freely accessible at https://github.com/profeatmap/ProFeatMap under the GPL license.

**Supplementary information:**

Supplementary data are available at *Bioinformatics Advances* online.

## 1 Introduction

Most omics studies produce datasets involving substantial lists of proteins. A useful approach for examining such lists is to analyze either their protein-associated biological properties as by Gene Ontology (The Gene Ontology Consortium, 2019) or their sequences. While Gene Ontology might be able to find over-represented features in the protein list, it loses information of their relative location, size and organization. Alternatively, sequence examination implies complex analyses as multiple alignments and requires similar proteins to be informative. An intermediate scale of analysis is to focus on features such as domains, amino acid or domain repeats, post-translational modifications, sequence variants, secondary structures, low-complexity regions and their organization along the sequence. Several online tools are already available to depict domain organization in a manual or semi-automatic manner (Table 1). However, even the most versatile programs, such as MyDomains on Prosite (Hulo et al., 2008), can only scrutinize one single protein at the same time, representing a tedious and time-consuming approach even for a list containing a limited number of proteins. The interactive Tree of Life (iTOL) tool bypasses this limitation, but specifically needs a phylogenetic tree as input (Letunic and Bork, 2021). The recent web interface CFVisual allows to plot domain organization for large protein datasets, but is still fed with DNA sequences (Chen, 2022). To our knowledge, a versatile tool allowing to visualize annotated features for large protein datasets in both a global and customizable way is still awaited. The use of lists of unique and simple identifiers such as the Uniprot entries rather than tedious protein or DNA sequences would be highly beneficial for this purpose.

**Table 1.** Overview of different online tools including ProFeatMap, that can be used for 2D protein representations, and comparison of their main properties

	UniProt	Interpro^a^	Pfam^b^	Prosite	SMART^c^	CFVisual	iTOL	ProFeatMap
Input type					Query	GFF3, GTF, BED	Tree	Protein list

Multiple proteins					✓	✓	✓	✓

Simple 2D view			✓	✓	✓	✓	✓	✓

Customisation				✓		✓	✓	✓

Savable modifications						✓	^d^	✓

Editing in UI				✓		✓	^d^	✓

Savable images		✓	✓	✓	✓	✓	✓	✓

Showing values				^e^			^e^	✓

Web interface	✓	✓	✓	✓	✓		✓	✓

a Blum (2021).

b El-Gebali (2019).

c Letunic and Bork (2018).

d Options only accessible with an account.

e Limited possibility.

Here, we introduce ProFeatMap, a freely available, user-friendly and interactive web interface. Gathering annotated features directly from well-established databases such as Uniprot (The UniProt Consortium, 2021), the program represents the overall domain organization along the sequence and many other features in a 2D map in which one dimension corresponds to the different proteins contained in the list, and the second dimension to their size. ProFeatMap proposes default parameters for a 1-click run, or advanced options allowing users to produce specific plots tailored to their needs. ProFeatMap is a powerful tool for overviewing at a glance a large protein set and gaining further insights into their common or distinctive properties.

## 2 ProFeatMap implementation

ProFeatMap is entirely coded in Python (3.9.6). It is freely accessible as a web interface developed using the Dash library (2.0.0), making it compatible with most common web browsers. Maps and corresponding legends are drawn with the pillow library (7.2.0). The program can be run through a website without any account or registration. Alternatively, all scripts are freely accessible on GitHub and can be run locally.

ProFeatMap follows four main steps: protein list uploading, features extraction, addition of user numerical values (optional) and map creation and customization. A user guide for each step is found in the help sections of the web interface. A detailed user guide is available within the Supplementary Information.

According to a user-defined protein list, ProFeatMap automatically downloads the UniProt file for each protein (https://uniprot.org), then extracts the protein features and subsequently determines their occurrences over the entire protein set. Several pieces of information are gathered: features with starting position and length, occurrence, a list of all the associated PDB files, amino acid sequences and sequence lengths. Alternatively, a different database can be selected among InterPro, SMART, Pfam, in which case the Uniprot domains and repeats will be replaced by the annotations of the selected database.

## 3 The ProFeatMap usage

The user provides a protein list as a table file (xlsx, xls, ods, csv, tsv and txt) containing the UniProt Accession codes and the optional unique protein identifiers. Alternatively, a compatible list can be obtained from UniProt by downloading a protein selection through a query or the basket option. Entry lists may also be composed of the UniProt accession codes of various proteins obtained by experimental omics approaches. After extracting feature occurrences, ProFeatMap generates a highly customizable 2D map. By default, ProFeatMap sorts the proteins based on the occurrences of domain, repeat, region and motif features. The number of similar features leads to a similarity value, while the number of differences gives a dissimilarity value. A first sorting by ascending order of the dissimilarities is done, followed by a second sorting by descending order of the similarities. This process results in transitions between different groups of highly similar proteins. It is particularly useful when dealing with large lists of proteins as it likely associates proteins with similar architectures even without any other prior knowledge.

The full process can be done through a semi-automatic run in a single click (called one-click run) or by adjusting one or several options (called customized run).

### 3.1 One-click run

**Fig. 1.**
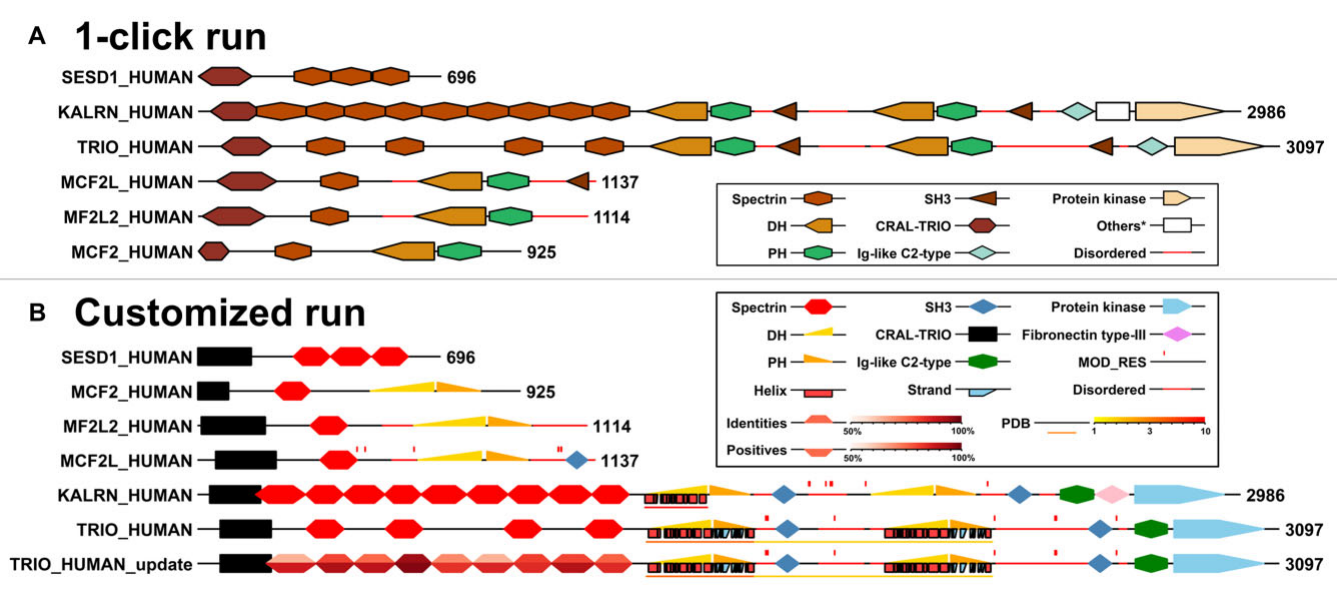
Examples of ProFeatMap outputs. (**A**) Map generated using the default parameters through the one-click run for proteins closely related to the Trio protein. The one-click run is useful to quickly visualize feature organization without *a priori* consideration. (**B**) Map obtained for a run performed for the same protein list when combining most of the options available. Disordered regions, structural coverage (PDB), secondary structure (Helix, Strand), compositionally biased regions (COMPBIAS) and post-translational modifications (MOD_RES) options are activated. While Kalirin and Trio are highly similar proteins, Uniprot considered nine spectrin repeats for Kalirin, but only four for Trio. The sequences of Kalirin and Trio were aligned with Blast, allowing to identify the five missing spectrin repeats in the Trio protein, that were subsequently added in the feature list and displayed in this map as ‘TRIO_HUMAN_update’. The proposed spectrin repeats are colored according to sequence identity and similarity percentages obtained from the two aligned sequences

In a one-click run, according to a user-defined protein list, ProFeatMap automatically downloads, extracts and maps feature data using all default parameters. It randomly assigns a combination of color and shape to each of the most frequent features displayed on the 2D map (Fig. 1A). The resulting map helps provide a global view of the ensemble of features. Such a one-click run is preferentially used for *ab initio* lists obtained for instance in the context of omics data. To even better appreciate the advantages of ProFeatMap, an example generated for human kinases (with 489 proteins) is visible on Supplementary File S1. Supplementary File S2 allows the user to compare it with the map generated by a one-click run for the PDZ list provided as an example in Step #1.

### 3.2 Customized run

Several toggle options are proposed to display features in the protein map, including disorder or structural information and coverage, as well as the positions of low-complexity regions or post-translational modifications. Geometric shapes and colors of the features are also fully customizable. Finally, selected features can be colored according to numerical values. These could be any quantitative or qualitative values, either extracted by data mining as the number of ligands, percentage of sequence homology, reads or publications related to a given domain, or obtained experimentally as affinities (Fig. 1B). Proteins in the map can be sorted based on those values.

### 3.3 Additional usages

ProFeatMap may help identify recurring feature patterns in proteins. An incomplete pattern might be indicative of annotation issues in the protein sequence database. Subsequently, the user may add or remove feature elements collected by further investigation directly in the web interface within the Step #2 of ProFeatMap.

ProFeatMap also allows extracting all the sequences of a given feature in *fasta* format or searching for motifs using regular expressions.

### 3.4 Local installation

The web interface of ProFeatMap is well suited for users with lists of up to 500 proteins. For larger lists or more intensive usages of ProFeatMap, we highly recommend making a local installation. This not only allows lists of several thousands of proteins to be handled, but also gives live feedback on progression or potential errors. Changing the memory parameter also makes it possible to keep progress between steps in your web browser’s memory. Saving and uploading all intermediate files every time the tab is closed would therefore not be necessary anymore. More information on how to install and change this parameter can be found in the user guide.

## 4 Conclusions

ProFeatMap is a powerful and highly customizable tool to quickly create publication-quality maps displaying domains and other key features along the protein sequences for a set of proteins of interest, and potentially including quantitative or qualitative data. Compared with some conventional motif visualization tools, ProFeatMap does not need nor use any sequence-based predictions. It merely uses feature information accessible in potentially several protein databases. The general overview provided by the representation helps gaining valuable insights into feature organization. The advantages and versatility of ProFeatMap can be even better appreciated when considering either a set of proteins with common features in a unique species, or a set of a particular protein originating from several species. Additionally, it is also suitable for the identification of characteristic elements such as domain patterns, which could prove to be of interest for the project.

## Supplementary Material

vbad022_Supplementary_Data
