# Supplementary material for: ProFeatMap: a highly customizable tool for 2D feature representation of protein sets
Source: Bioinform Adv. 2023 Mar 9;3(1):vbad022. doi: 10.1093/bioadv/vbad022 (PMC10023195; doi:10.1093/bioadv/vbad022)
Supplement: vbad022_Supplementary_Data [file vbad022_supplementary_data.zip › User_guide_v1.3.2.pdf]

User Guide

Description: ProFeatMap is an online web interface allowing to create highly customizable 2D representations of protein lists based on Uniprot data.

Written by: BICH Goran

Contact information: profeatmap@gmail.com

Last update: January 20, 2023

ProFeatMap version: 1.3.2

Summary: ProFeatMap creates 2D representations (maps) of elements of interest (features; e.g. domains, repeats, disorder, post-translational modifications, etc) for a list of proteins based on the information available in the Uniprot database following several steps:

- Step 1: The user provides a list of proteins as Uniprot accession codes that ProFeatMap will use to download all their associated Uniprot files from the Uniprot database.
- Step 2: Extract the features from the downloaded files, compiling data in a single output file that can be downloaded by the user
- Step 3: An optional step to add numerical values that can be displayed with colormaps in Step 4.
- Step 4: Creation of the map itself.

Abstract:

Motivation: Studies of sets of proteins are a central point in biology. In particular, the applications of omics over the past decades have produced lists of several hundreds or thousands of proteins or genes. However, these lists are often not inspected globally, possibly because of the lack of tools capable of simultaneously visualizing the feature architectures of a large number of proteins.

Results: Here, we present ProFeatMap, an intuitive Python-based website. For a given set of proteins, it allows to display features such as domains, repeats, disorder or post-translational modifications, and their organization along the sequences, into a highly customizable 2D map. Starting from a user-defined protein list of UniProt accession codes, ProFeatMap extracts the main annotated features available for each protein from one of the well-established databases such as Uniprot or Pfam, allocates shapes and colors, potentially depending on quantitative or qualitative data, and sorts the protein list based on homologous feature content. The resulting publication-quality map allows to investigate even large protein families, and to classify them based on shared features. It can help to gain insights, e.g. feature redundancy or feature pattern, that were previously overlooked. ProFeatMap is freely accessible on the web at: https://profeatmap.pythonanywhere.com/

Table of content

I. General comments 5

I.1. Description 5

Feature 5

Map 5

I.2. Components 5

I.2.a. Drag and Drop or Select Files 5

I.2.b. Tables 6

I.2.c. Downloadable files 6

II. Quick run guide 6

II.1. 1-click run 6

Run to create only the map and the legend using default parameters. After uploading a list of proteins (Step 1), click the 1-click run button. The map is directly displayed at the bottom of Step 1 section using default options with very limited control. The 1-click run is best adapted for protein lists up to 200 proteins. 6

II.2. Customized run 6

III. Step 1: Protein data gathering 7

III.1. Description 7

III.2. Components 7

III.2.a. Protein list 7

III.2.b. Remove organism 8

III.2.c. Database selection 8

IV. Step 2: Feature extraction 8

IV.1. Description 8

IV.2. Components 8

IV.2.a. Modification file (optional) 9

IV.2.b. Structural coverage extraction 9

IV.2.c. Feature sequence extraction 10

IV.2.d. Feature/Motif search by regular expression 10

IV.2.e. Extract only 10

10

V. Step 3: Numerical values addition 10

V.1. Description 10

V.2. Components 10

V.2.a. Numerical values table 10

11

VI. Step 4: Map creation 11

VI.1. Description 11

VI.2. Components 11

VI.2.a. Shapes and colors table 11

VI.2.b. Protein cuts table 12

VI.2.c. Automatic feature selection 12

VI.2.d. Sorting 12

VI.2.e. Value related 13

VI.2.f. Feature parameters 13
VI.2.g. General feature parameters 13
VI.2.h. Order of feature drawing 14
14

VII. Local installation **14**
VII.1. When is it advised? 14
VII.2. How to? 14
VII.3. Storage type: memory vs local 14

I. General comments

I.1. Description

Feature

A feature is an element of interest in a protein. These features can be domains, repeats, post-translational modifications, variants, secondary structure,… These features appear in the “FT” category in Uniprot files.

Map

In ProFeatMap, a map is a schematic 2D representation of proteins in which features are represented according to their relative position and size on a given protein.

I.2. Components

I.2.a. Drag and Drop or Select Files

<u>Description</u>

ProFeatMap allows the user to upload files in several formats. It can be done either by drag and drop or by file selection. The same file formats are also recognized for files without extension.

<u>Compatible file formats</u>

“***.xlsx***”: 2007 and later Excel version file format.
“***.xls***”: Excel file format before 2007.
“***.ods***”: LibreOffice Calc file format.
“***.csv”, “.tsv”, “.tab”, “.txt***”: These file formats are also recognized by ProFeatMap.
Separators will be considered by descending priority order :
- tabulations
- “;” character
- “,” character

<u>Warnings</u>

Numerical values in columns should not be formulas and decimal separators should be “.”.

I.2.b. Tables

<u>Description</u>

Tables allow providing ProFeatMap with information needed to process the different steps. There is a total of 5 tables in the interface:

“***Protein list***”: (*mandatory*) This table contains the list of Uniprot accession codes of the proteins to be represented on the map.
“***Modifications***”: (*optional*) This table contains modifications to be done during the extraction. This table is used to “correct” data or to manage more precisely the display of the proteins.
“***Numerical values***”: (*optional*) This table contains the numerical values given to specific features of the proteins. These could be any quantitative or qualitative values, either extracted by data mining as the number of ligands, percentage of sequence homology, reads or publications related to a given domain, or obtained experimentally as affinities.
“***Shapes and colors***”: (*optional*) This table contains the display parameters of each feature for fine tuning of the map creation. The VI.2.c. Automatic feature selection option fills up the table automatically and can be used as a starting point. If the table is empty, the table will be filled automatically using default parameters.
“***Cut regions***”: (*optional*) This table contains regions of proteins that should be hidden on the map.

<u>File name</u>

The name of the files is up to the user, only the content is used by ProFeatMap.

Mandatory columns

Each table has a number of columns that is recognized by ProFeatMap. Therefore, it is important to make sure all these column names appear in the tables. Warning: Column names are case sensitive.

Additional columns

When building these tables, additional columns can be added. Names of additional columns must be unique, and distinct from those of mandatory columns. Spaces and special characters in the names should be avoided.

Buttons

Resets all map parameters with the default values.

Adds a row at the end of the table. Empty rows should be avoided. The new table has to be saved ( ) to be taken into account.

Used to completely clear the table, leaving only the template columns and an empty row. Clearing a table needs to be saved ( ).

Used to save changes made in the table. Unsaved modifications will not be taken into account when executing the next step. This button will automatically be highlighted ( ) when modifications are made and switched off when clicked.

Download the latest saved table as an .xlsx file. If no table is displayed, a template file will be provided with headers only.

I.2.c. Downloadable files

Description

ProFeatMap allows the user to locally download different types of files depending on each step.

Formats

"***.xlsx***": Table, extracted data and map parameters can be downloaded as Excel files.
"***.fa***": In Step 2, sequences of a feature can be extracted. The result can be downloaded as a .fa file compatible with multiple alignment tools.
"***.png***": The maps and legend generated by ProFeatMap can be downloaded by right clicking and saving images in .png format.

II. Quick run guide

II.1. 1-click run

Run to create only the map and the legend using default parameters. After uploading a list of proteins (Step 1), click the 1-click run button. The map is directly displayed at the bottom of Step 1 section using default options with very limited control. The 1-click run is best adapted for protein lists up to 200 proteins.

II.2. Customized run

This section shows an alternative to create a map by following 5 simple steps. It guides new users through the main process of creating maps while being able to customize them.

1. Upload a protein list in the Drag and Drop zone of Step 1 section (Two examples files of protein list can be downloaded for testing, TRIO and PDZ domain families).

2. Click on the Customized run button and wait until finished.

3. Go to Step 2 section and click the Run ▶ button.

4. Use Step 3 if numerical values are available for some features to be displayed. Otherwise, skip Step 3 and go directly to Step 4 (see “V. Step 3: Numerical values addition")

5. Click the Run ▶ button in Step 4. The map will be shown once generated (see “VI. Step 4: Map creation” if you want to customize your map).

III. Step 1: Protein data gathering

III.1. Description

The user provides ProFeatMap with a list of proteins to appear in the map. This list contains Uniprot Accession codes (mandatory) and names for the proteins (optional). Subsequently, ProFeatMap downloads the Uniprot files corresponding to the provided codes.

III.2. Components

III.2.a. Protein list

Table construction

“***code***” or “***Entry***”: Column containing Uniprot accession codes corresponding to the protein. The same code can be used multiple times.

“***protein***” or “***Entry name***” (optional): Column containing the names of the proteins that will be used during all the process. It has to be unique and will serve as an identifier to ProFeatMap. ProFeatMap will automatically search for Uniprot names after downloading files if names are missing and fill the table accordingly.

Notes

The header is not mandatory. If absent, ProFeatMap will automatically consider the first column as the “code” column. If only one more column is given and no duplicates are found in the column, the column is considered as the “protein” column. Otherwise, all additional columns are discarded.

If only codes are given as a list, names of proteins will be automatically added to the table after retrieving data from Uniprot. In case of duplicated Uniprot codes, a duplication tag will be added to the protein name to avoid indexing problems.

Uniprot search compatibility

Selected proteins of interest or basket can be exported directly from the Uniprot webpage as a compatible Excel file. “Excel” and “Uncompressed” must be selected, as shown below.

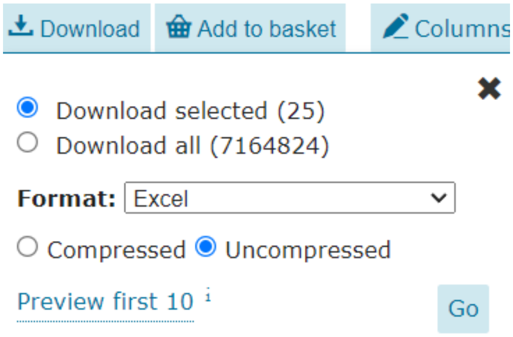

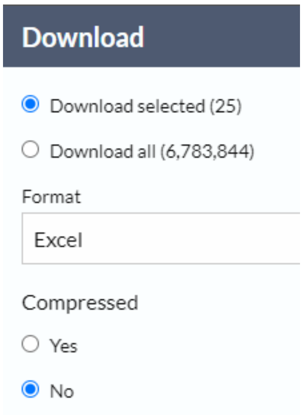

III.2.b. Remove organism

Description

This option removes the organism tag from the protein names in the list (i.e. TRIO_HUMAN will be transformed into TRIO). It only works if all protein names have the same tag. This feature is meant to be used on protein names originating from Uniprot, i.e. for users interested in a single organism.

Downloading troubleshooting

If the protein list contains obsolete or invalid Uniprot codes, a section with a downloadable file containing these codes will appear. More information on the problem can be obtained by searching for the incriminated codes on Uniprot. The codes should then be replaced or removed.
If the whole protein list shows up in the file, it is most likely caused by a formatting error. Alternatively, a temporary loss of connection to the Uniprot database would have the same result. The current state of the connection can be obtained by refreshing ProFeatMap webpage and check the status next to the Uniprot website link:

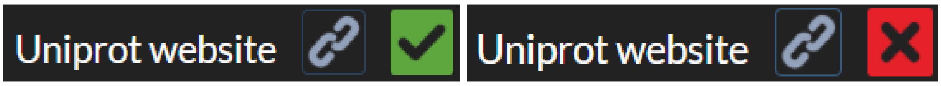

III.2.c. Database selection

Description

ProFeatMap uses Uniprot data by default. However, it is also possible to retrieve data coming from other databases (Interpro, SMART and Pfam). Choosing any database which isn't Uniprot will do the following: Uniprot data is retrieved as by default, but all features categorized as DOMAIN or REPEAT are removed and replaced by the annotations of the selected database.

Parameters

"***Database***": (*default: uniprot*) Choice of the database to use for features.

"***Replace mode***": Certain databases contain domains, homologous superfamilies or both. This option is used to select which kind of annotation should be used. Most of the databases have only one category, selected by default and act as information for the user.

IV. Step 2: Feature extraction

IV.1. Description

The main goal of this step is to extract features found in the Uniprot data files ProFeatMap gathered in Step 1. Additionally, a modification table to add or remove features can be provided. This step also contains additional tools: feature sequence extraction and feature/motif search by regular expression.

IV.2. Components

IV.2.a. Modification file (optional)

Description

Table filled with features to add or remove during the extraction process.

Accessibility

Through the Modification options button.

Table construction

"***ex_type***": Column filled to add ("add" or "+") or remove ("remove" or "-") a feature.

"***protein***": Name of the protein to modify.

"***feature_type***": Column to indicate the type of feature (DOMAIN, REPEAT, HELIX, BINDING, ZN_FING...). When removing a feature, the corresponding type can be checked in the output extracted data file. Not specifying the type will result in removing all features with the feature name which should be fine with most applications if not all. When adding a feature that already exists, using the same feature_type should be considered. For the addition of a not yet existing feature, DOMAIN can be used. Using other feature_type may be used for specific uses such as drawing order of features in the final map. You do not need to specify the type, in which case it will be categorized as DOMAIN by default.

"***feature***": Column to indicate the name of the feature. This name will appear on the legend.

"***start***": Starting position of the feature. While removing, if this parameter isn't specified, all occurrences of the feature in the protein will be removed.

"***length***": Length of the feature. While removing, if this parameter isn't specified, all occurrences of the feature in the protein will be removed.

IV.2.b. Structural coverage extraction

Warning

This option is not accessible anymore since v1.3.2.

<u>Description</u>

ProFeatMap searches for resolved 3D structures and calculates the number of structures available in each protein. This "coverage" can be displayed in Step 4.

<u>Accessibility</u>

Through the Modification options button.

<u>Parameters</u>

"***Step number***": (*default*: 5) The colormap is cut into a discrete series of colors. The number of resulting colors is given by the Step number. See example below.

"***Colormap thresholds***": (*default*: [1, 2, 3, 5, 10]) Each color from the discrete colormap is associated to a threshold value corresponding to the number of resolved 3D structures needed to be represented with the associated color. The color of the first step is the color at 0.2 on the base colormap. If there is only one step, the color will be the color at 1 on the base colormap. Number of structures below the first number in the array (1 in the default), will not appear on the final map.

<u>Examples</u>

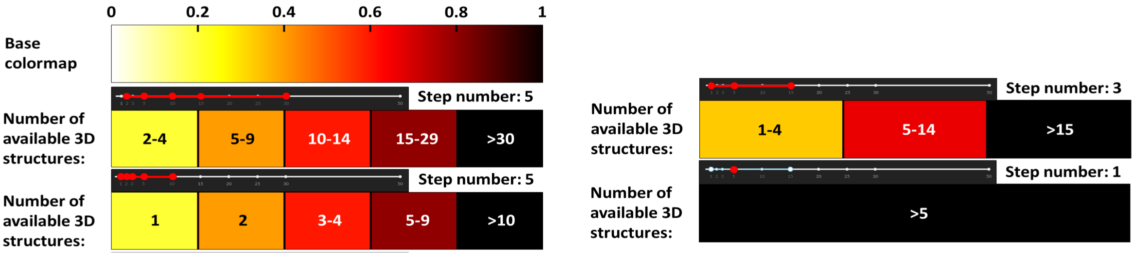

IV.2.c. Feature sequence extraction

<u>Description</u>

Optional tool used to get the sequences of all occurrences of a given feature in the whole protein list. This tool will only appear once Step 2 has been run at least once.

<u>Accessibility</u>

Through the Advanced extraction options button after running Step 2.

<u>Parameters</u>

"***Feature name***": The name of the feature that must correspond to the name found in the extracted data file. The name is case sensitive. With the "N-ter" and "C-ter" parameters set to 0, ProFeatMap will extract the sequences as defined in Uniprot.

"***N-ter ext***": (*default*: 0) Positive values will extend the sequence by the value towards the N-terminus of the protein. Negative values will shorten the sequence towards the C-terminus.
"***C-ter ext***": (*default*: 0) Positive values will extend the sequence by the value towards the C-terminus of the protein. Negative values will shorten the sequence towards the N-terminus.

IV.2.d. Feature/Motif search by regular expression

Description

Optional tool which can be used to find the localization of features or motifs based on a regular expression. The output file can be used as a modification file to add the found features during the extraction step. This tool will only appear once Step 2 has been run at least once.

Accessibility

Through the Advanced extraction options button after running Step 2.

Parameters

"***Feature regular expression***": To make the search needed to build a regular expression of the feature or motif. More info on how to build a compatible regular expression here: https://docs.python.org/3/library/re.html or build it directly here: https://pythex.org/
"***Feature name***": The name to give to the extracted feature/motif. This is the name that will appear on the final figure if output is added to the modification table.

IV.2.e. Extract only

Description

Downloads the extracted data file directly after finishing Step 2 without storing the result of the extraction in the user's web browser. This option is meant to be used in case of a large list of proteins (>1000), where ProFeatMap will most likely not be able to save the extracted data on the browser because of the lack of memory space. Activating this option will however not allow you to make further steps nor create a map.

Fix options

Emptying your browser's navigation data may free enough space to be able to store the extraction. Reducing the size of the protein can be an option. If these two solutions are not working, is it always possible to install ProFeatMap locally and change the storage_type='local' to storage_type='memory' in the app.py script (See "VII. Local installation" for further details).

V. Step 3: Numerical values addition

V.1. Description

This section shows how to add numerical values to features that can then be displayed on the map using a color scale.

V.2. Components

V.2.a. Numerical values table

Description

A table containing a list of features and the numerical values that should be associated.

Table construction

"***protein***": name of the protein that contains the feature.
"***feature***": name of the feature. This name has to correspond to the exact name of the feature that appears in the extracted data file.
"***start***": the starting position of the feature. This value has to appear in the extracted data file. It is used to identify the feature (if multiple).

"*condition_x*": multiple conditions can be added. One column per condition is needed and must have a unique user defined name. Spaces should be avoided. These conditions can then be selected in Step 4 for the map creation.

Values

The values given by the user should be normalized (between 0 and 1). -1 will be interpreted as missing value and will appear in gray on the map. No value will result in the use of the specified "color" and "contour_color" in the map Shapes and colors table.

VI. Step 4: Map creation

VI.1. Description

Creates the map of the protein list using shapes and colors either defined automatically or defined by the user potentially considering cut regions. Map parameters are used to change the overall map generation, the display of specific features, the order of feature drawing and the condition (if any) to show numerical values.

VI.2. Components

VI.2.a. Shapes and colors table

Description

The shapes and colors table contains the list of features to appear as specific shapes and colors on the protein map. This table will be filled using the Automatic feature selection with default parameters if empty.

Accessibility

Through the Feature display options button.

Table construction

"***shape***": shape of the feature. A list of available shapes can be found below the table.
"***orientation***": some shapes have associated orientation that have to be specified.
"***height***": (*default*: vertical stretch factor) corresponds to the height, in pixels of the feature.
"***contour_color***": (*default*: black) color of the contour of the shape.
"***contour_colormap***": (*default*: none) used to show numerical values on the contour of the shape. If the Uniform shape fill/contour is selected, a "colormap" is defined and no "contour_colormap" is selected, the edges share the "colormap" with the inside of the shape.
"***contour_threshold***": (*default*: none) Only used if a "contour_colormap" is defined. A value between 0 and 1 can be indicated. Values below this threshold will appear in the color defined in "contour_color". Values above the threshold will use the "contour_colormap".
"***color***": (*default*: white) color used to fill the shape.
"***colormap***": (*default*: none)
"***threshold***": (*default*: none) Only used if a colormap is defined. A value between 0 and 1 can be indicated. Values below this threshold will appear in the color defined in "color". Values above the threshold will use the "colormap".
"***pensize***": (*default*: protein thickness) corresponds to the thickness of the contour of the feature.

Showing other features

Adding "Others" as a feature name will show all features that are not listed in the table using a white rectangle with black contour by default.

VI.2.b. Protein cuts table

Description

To shorten specific proteins by hiding a fraction of the protein (indicated by a "//" on the map) and all included features.

Accessibility

Through the Feature display options button.

Table construction

“***protein***”: Name of the protein to be cut out.
“***start***”: Starting position of the region to hide.
“***length***”: Length of the region to hide.

VI.2.c. Automatic feature selection

Description

Automatic feature selection will search in the extracted features either the most represented features or features that appear more than a given occurrence in the list. Only features under the DOMAIN, REPEAT, MOTIF or REGION tag are considered. If the feature is one of the most common ones (DOMAIN and REPEAT in the human proteome), the default shape and color will be used. Other features will have a random shape and color. All features that are not represented enough will be represented by the “Others” category. By default “more than 2 occurrences” is used.
It will automatically fill Shapes and colors tables when empty using default parameters.

Parameters

“***x most represented features***”: (*default*: square root of the number of proteins) automatic feature selection will choose a shape and color for the top x most represented features
“***more than x feature occurrences***”: (*default*: 2) automatic feature selection will choose a shape and color for features that have been found at least x times in the protein list

Lock seed

This option can be toggled to fix the current seed used for random picking of shapes and colors by the automatic feature selection.

VI.2.d. Sorting

Description

ProFeatMap features several sorting options impacting the order of appearance of the proteins on the map.

Options

“***None***”: order of the proteins as defined in the table in Step 1.
“***abc***”: to sort the protein list in alphabetical order.
“***feature_number_distance***”: (*default option*) to let ProFeatMap sort automatically the proteins by gathering those with similar feature content. WARNING : This sorting is quite intensive. It should not be used with large lists of proteins (>1,000). The resulting sorting can be saved with the “Latest sorting protein list” button. This list can be used as input list, and None put as sorting. This way of proceeding is strongly advised for lists with more than 200 proteins in order to decrease the drawing process time.
“***value***”: see VI.2.e. Value related below.

Feature number distance

By default, ProFeatMap sorts the proteins based on the occurrences of domain, repeat, region and motif features. The number of similar features leads to the similarity value, while the number of differences gives the dissimilarity value. A first sorting by ascending order of the dissimilarities is done, followed by a second sorting by descending order of the similarities. This process results in transitions between different groups of highly similar proteins. It is particularly useful when dealing with large lists of proteins as it likely associates proteins with similar architectures without any other prior knowledge.

VI.2.e. Value related

Description

Sorting by value is only possible if the user has given numerical values in Step 3. This sorting will order proteins by descending values. Each protein is represented by the highest value if multiple occurrences of the target feature is found.

Parameters

"***Case to draw***": (*default*: None) all conditions defined in the numerical value file will appear in this list when Step 3 is run. Selecting a condition will affect the values displayed on the map.
"***Focus on***": (*default*: None) the name of the feature by which the map should be sorted must be inputted here.
"***Threshold***": (*default*: None) by indicating a threshold (float value), allows to remove from the map all proteins focused on features with values below the threshold.

VI.2.f. Feature parameters

Description

These parameters can be toggled on or off to make specific features appear or disappear from the created map.

Parameters

"***3D structure coverage***": (*default*: no) As defined during extraction (see Structural coverage extraction), toggling this option will show a line where resolved 3D structures have been found in the PDB database. The line is colored depending on the number of structures.
"***Secondary structure***": (*default*: no) Secondary structures (helixes, strands and turn) will be represented on the proteins.
"***Disorder***": (*default*: yes) When toggled, predicted disordered regions will be shown on proteins.
"***Modified residues***": (*default*: no) When toggled, modified residues (phosphorylation, glycosylation, …) will appear on proteins.
"***Composition biased regions***": (*default*: None) Biases can be selected, and will appear on the proteins.

Feature parameters default

Clicking this button will add to the current shapes and colors table the default representation of each selected "Feature parameter" in the "Map parameters". The addition of these representations will overwrite the default representation. This will also cause these parameters to be always displayed whenever the corresponding parameter is toggled or not.

VI.2.g. General feature parameters

Description

General feature parameters will affect general visual aspects of the map such as horizontal and vertical stretch, protein thickness, text sizes and the display of the protein length.

Accessibility

Through the Map options button.

Parameters

"***Figure width factor***": (*default*: 1) multiplying factor of the horizontal width of the map canvas > 1 will make a bigger map, whereas < 1 will make it smaller. Support floats. The default width is 4,000 pixels.
"***Horizontal stretch factor***": (*default*: 1) multiplying factor of the horizontal length on the map > 1 will cause the proteins and features to appear longer, whereas < 1 will make them appear smaller. Support floats.
"***Vertical height***": (*default*: 20) vertical space used by each protein. Increasing the value will make the figure bigger. It will also cause all features shapes that have no "height" specified to have this value as default value.
"***Protein thickness***": (*default*: 3) thickness of the line representing the protein. This value is the default pensize value for feature shapes (contours), if none is specified.
"***Protein name size***": (*default*: 30) size of the text showing the protein names.
"***Biased regions text size***": (*default*: 20) size of the text shown above the composition biased regions.

“***Show protein length***”: (*default*: yes) toggle to activate or deactivate the display of the protein length next to each protein.

“***Consistent shape fill/contour***”: (*default*: no) toggle to activate or deactivate the standardization of the filling color and the contour color. When activated, all feature shapes that have no “contour_color” specified, will appear the same color as the filling color (if there is one). It also applies to colormaps.

VI.2.h. Order of feature drawing

Description

This list of feature categories defines the order in which the features will be drawn on the proteins. In case of overlapping features, the category appearing the latest in the list will appear above. Default parameters should avoid most overlaps.

Accessibility

Through the Map options button.

Parameters

(*default*: DISORDER, DOMAIN, CHAIN, INIT_MET, ZN_FING, DNA_BIND, REGION, ACT_SITE, METAL, SITE, LIPID, HELIX, STRAND, TURN, CONFLICT, CARBOHYD, BINDING, MOTIF, MOD_RES, COMPBIAS, REPEAT, VARIANT, PDB)

VII. Local installation

VII.1. When is it advised?

A local installation of ProFeatMap is advised for maps of protein lists larger than 500 proteins or more intensive usage. This will get rid of the 5 minutes time limit for each step imposed by the web interface and also allows keeping processing data into your web browser if desired. A local installation will also help to provide more feedback on bugs.

VII.2. How to?

Copy ProFeatMap from the Github repository: https://github.com/profeatmap/ProFeatMap

Install Python and the libraries needed for ProFeatMap. The Python and libraries versions can be found in the main script “app.py”. The libraries can be installed in the same order as found in the script.

(optional) Change “storage_type='memory'” to storage_type='local' if wanted

Run “app.py” script.

Open a tab on your web browser and go to: http://127.0.0.1:8050/

VII.3. Storage type: memory vs local

ProFeatMap is in “on-the-fly” mode on its website form. This means that data between steps are not kept in the browser's memory and everything will be reset by refreshing the page. The advantage is a more user-friendly usage for new users. This will also avoid storage capacity issues.

ProFeatMap in “local” mode is more suited for intensive usage, as it will keep progress in memory, even after refreshing the page, or opening again the webpage. This will avoid having to save progress and/or upload every file every time. Last map, legend, extracted data, and tables will be directly accessible. This also allows erasing unwanted and unsaved modifications in the UI tables by refreshing the page. This can however be confusing for new users. There is a limit to the protein list size stored in the browser which can cause ProFeatMap to not be able to work properly for lists in the several thousands of proteins. For proteome sized lists, please use the “local” mode.
